# The Vaccine Treatment of the Common Cold

**Published:** 1910-06-04

**Authors:** 


### Medicine.

### THE VACCINE TREATMENT OF THE COMMON COLD.

That very great benefit is to be derived from the vaccine treatment of the common cold, especially in patients who are susceptible to catching cold, is well exemplified by the account given by a patient of the results of treatment in his own case, as described in a letter to the *Lancet*. For nearly twenty years the sufferer had been a martyr to catching catarrhs at the slightest provocation; almost every change of weather affected him. His trouble almost invariably began in the post-nasal space and within a short time spread to the larynx and trachea. He usually had a copious secretion from the nasal mucosa and the catarrh then spread to the Eustachian tubes. So troublesome were these symptoms that he had frequently had the turbinate bones and the post-nasal space cauterised in the hope of improvement, but without any benefit either from this or from any other form of treatment. He could never sit before an open window, or be in the slightest draught, or attend a night call without the inevitable cold coming on. As he himself said, no one could have suffered more or more continuously from the annoying chills than he did. A culture was made from a mixed nasal and laryngeal secretion, which showed:—

| | |
|---|---|
| Micrococcus catarrhalis ... ... | 75 millions |
| Bacillus influenzæ ... ... ... | 25 „ |

As this and subsequent injections were given at about 6 p.m., the after-effects of one will answer for a description of the rest. He had a commencing cold when the first injection was given, and about 3 a.m. he awoke with a headache and some feverishness. After three or four hours this passed off and during that afternoon the cold had wonderfully cleared up, though the trouble usually lasted for a week or two. Thereafter the following injections were given at the dates mentioned:—

| | | |
|---|---|---|
| July 2, 1909 | M. catarrhalis ... ... | 75 millions |
| | B. influenzæ ... ... | 25 „ |
| July 20, „ | Repeated as for July 2, 1909 | |
| Aug. 12, „ | M. catarrhalis ... ... | 250 „ |
| | B. influenzæ ... ... | 100 „ |
| Sept. 2, „ | M. catarrhalis ... ... | 375 „ |
| | B. influenzæ ... ... | 150 „ |
| | B. septus ... ... ... | 125 „ |
| Sept. 15, „ | Repeated as for Sept. 2, 1909 | |
| Sept. 27, „ | | |
| Oct. 12, „ | | |
| Nov. 2, „ | | |

No more injections were then given until February 17, 1910, when the following was given:—

| | |
|---|---|
| B. influenzæ ... ... ... ... | 250 millions |
| M. catarrhalis ... ... ... | 500 „ |
| Pneumococcus ... ... ... | 100 „ |
| B. septus ... ... ... ... | 125 „ |

Up to February 17 not one cold developed during the winter, and the slight one coming on then was quickly cut short by the last injection. In reality, then, the injections gave four months of complete immunity; the patient writes saying he has had no injection since February last and no cold, and the great comfort and improvement experienced through the treatment impels him to record his own personal sense of the great benefits of vaccine therapy to those who are susceptible to catching cold.

